# Surgeon accountability for patient safety in the Acute Care Surgery paradigm: a critical appraisal and need of having a focused knowledge of the patient and a specific subspecialty experience

**DOI:** 10.1186/s13037-015-0084-3

**Published:** 2015-11-13

**Authors:** Salomone Di Saverio, Gregorio Tugnoli, Fausto Catena, Arianna Birindelli, Carlo Coniglio, Giovanni Gordini

**Affiliations:** Maggiore Hospital Regional Emergency Surgery and Trauma Center – Bologna Local Health District, Bologna, Italy; Maggiore Hospital of Parma, Parma, Italy

**Keywords:** Postoperative morbidity, Continuity of care, Specificity of expertise, Surgical postoperative complications, Risk of mortality after major surgery, Outcomes, Consultant-led surgical service, Emergency surgery management and outcomes, Surgical ward-care checklists, Dedicated acute surgical admission ward

## Abstract

There is an increasing evidence in the literature showing that Acute Care surgical patients, likewise patients from every other surgical subspeciality, should be best first approached and managed only by attending surgeons with approriate expertise in the field of Emergency and Trauma Surgery, as well as the occurrence of postoperative complications can be prevented or safely and appropriately treated when arising, only by those attending surgeons having a focused knowledge of the patient and specific subspeciality experience. The advantages of a consultant-led, patient-centered surgical management come along with the opportunity of maintaining the principles of continuity of care and specificity of expertise in managing surgical patients and their complications and readmissions. These principles should be particularly valid in the well-recognized subspeciality of Acute Care and Trauma Surgery; managing the challenging emergency surgical patients either in the preoperative and postoperative periods with the aim to improve the outcomes of Emergency Surgery, should only be by surgeons trained and experienced in both Acute Care Surgery and Trauma.

The debate on the opportunity of prevention or of a safe and appropriate management of postoperative complications in surgical patients, has gained a considerable and increasing interest in the last months.

From our daily clinical experience in a surgical department of a western European country, gathering a wide variety of general surgery subspecialties (including HBP surgery and colorectal surgery and upper GI surgery as well as Emergency and acute care surgery, whereas Trauma Surgery service is most surprisingly separated from Emergency Surgery service) under only one chairman taking every final clinical decisions, where the patients are often managed in the ward by attending surgeons dedicated only to ward-round activities and where the hierarchical structure leads to decisions taken by somebody who was not the surgeon treating the patient in theatre and therefore having the necessary knowledge of the surgical case, we have commonly seen a raised incidence of inappropriate management of the postoperative complications. In the eyes of someone having different skills and subspecialty expertise, a postoperative leak or a post-operative ileus can become a challenging situation leading to inappropriately taking action too early or too late.This model of organization represents in our opinion a serious threaten to the safety of acute surgical patients. 

An additional serious challenge to patient safety in surgery has already been highlighted to reside in the questionable quality of training for the next generation of surgeons [1], ultimately leading to possible misjudgement and failure to rescue or to optimally manage postoperative complications in surgical patients.

After reading an interesting randomized trial from Pucher et al. [2], the results of this simulation-based study seems to suggest that use of Checklists resulted in significantly improved standardization, evidence-based management of postoperative complications, and quality of ward rounds. In addition checklists may represent a low-cost intervention to reduce rates of failure to rescue and to improve patient care.

It is notably true that the ward-based of surgical care is one rife with potential for adverse events and in every hospital we have seen mistakes and errors even from senior and experienced staff. It is also true that in management of postoperative morbidity, failures to adhere to principles of best practice, place patients at risk of avoidable harm and poorer outcomes, and as far we have seen in all countries we have been working, either in Italy, in our Hospital and Surgical ward, but also in Europe as well as overseas, there is a strong need for checklists guiding the doctors to the principles of best practice in an attempt to guarantee uniformity and homogeneity in postoperative care and in managing complicated surgical patients. Non-homogeneity in the assessment of postoperative complications and moreover in their need of going or not going back to theatre, errors in taking the most appropriate action in the postoperative management, mistakes or delay in critical clinical decisions, are a common and potentially preventable cause of increased significant morbidity and mortality and therefore of higher overall hospital and social costs. These otherwise avoidable errors are also carrying relevant medico-legal implications in the developed countries.

Although the results of this experimental simulation-based randomized trial are extremely interesting, it would be at least as interesting to see a clinical validation of this method in a real-life setting. Although the study setting was realistic and well reproduced, real life can still be rather different than simulation and see how the checklists works in a clinical environment would be a clinical validation of this method, perhaps within a further study. I would suggest the authors to discuss the possibility of designing a clinical study for validation of these surgical ward-care checklists.

We therefore believe that this study and the proposed checklists method to improve surgical ward-care, is overall interesting and beneficial, however of course in certain situations the doctors might need to think on their own and skip some steps of the checklists and be more straightforward and oriented to the problem solution (i.e. if major surgical bleeding occurs, resuscitation, theatre activation and senior help are definitely priorities over actions like crossing off the anti-thrombotics or ordering NBM to the nursing staff). In other words, sometimes the checklists may be dangerously distracting the trainees/ward doctors from life-saving actions such as going back to theatre for stopping the haemorrhage or taking back to theatre ASAP a patient with faecal discharge from an abdominal drain and diffuse peritonitis. All other actions within the checklists are useful and making sense but should not take the doctor’s attention away from life-saving decisions and from the main actions to be commenced.

Therefore every action within checklists should serve only as a general guide and must be taken with care, always keeping in mind what the appropriate priorities are in every clinical scenario or in different type of patients.

Another delicate issue in safety is the risk of inappropriate assessment management, either preoperatively or postoperatively, of the acute surgical patients subset, due to the potential lack of surgical critical care competence among the general surgeons [3].

Specifically speaking of the more delicate issue of assessing and admitting for observation the acute care patients from ED, potentially of surgical interest, we recall a recent study from Eijsvoogel et al. [4].

This is an extremely interesting pilot study addressing the innovative project of establishing an Acute Surgical Admission ward.

The idea of a step-down ward dedicated to short observation, for admitting and observing those patients coming from E.D. and believed to deserve a more careful assessment and a longer observation period is not new. These patients are deemed by the responsible physician to be able to go home with a reasonable short period of close observation and without need of a specialised ward admission, without the need of being admitted to a specialised ward.

These facilities for short observation are currently quite common in western countries for decreasing the overload of E.R. as well as of the specialised wards. This model of care is intended to be highly cost-effective and involve patients requiring a short course of investigation, observation or treatment, likely to take not less than 6 and no longer than 24–36 h. It is usually used without distinction for the assessment of either medical or potentially surgical patients. These facilities are designated with different names such as Clinical Decision Unit (CDU) in U.K. or O.B.I. in Italy (unit for Observation Brief and Intensive) or generically called ED observation units in U.S.

Eijsvoogel et al. have to be commended for their pioneering attempt to establish a similar model of care. The present study seems to be the first study reporting the preliminary results of such idea of a short observation unit only for surgical or potentially surgical patients.

Ideally the ASU should be able to decrease the proportion of patients admitted from ER to the specialised wards and therefore reduce the LOS, without negatively altering the rate of discharge from E.R. nor the readmission rate. ASU should be a way to shortly observe the patient who may potentially evolve in the next hours rather than being a parachute for the emergency physician, allowing a sub-optimal assessment of the patient (saving time and reducing the ER queue by moving patients to ASU).

In conclusion we believe that the advantages of a consultant-led and patient-centered [5] surgical management come along with the opportunity of maintaining the principles of continuity of care and specificity of expertise in managing the surgical patients and especially their complications and readmissions [6]. These principles are particularly valuable and advisable in the field of Acute Care Surgery and when managing the challenging emergency surgical patients both in the preoperative and postoperative periods, with the aim to improve the outcomes of Emergency Surgery [7].
